# Effects of boundaries for high Reynolds number artificial swimmers

**DOI:** 10.1038/s41598-025-99316-x

**Published:** 2025-04-24

**Authors:** Jean François Boudet, Michel Bergmann, Angello Iollo, Hamid Kellay

**Affiliations:** 1 University of Bordeaux, CNRS LOMA UMR 5798, Talence, F-33405 France; 2 University of Bordeaux, CNRS IMB UMR 5251, Equipe-project Memphis, Inria, centre de l’université de Bordeaux, Talence, F-33405 France

**Keywords:** Fluid dynamics, Applied mathematics

## Abstract

The spatial organization of active particles or swimmers may depend strongly on the nature of the interaction between the particles and the boundary. Here we use robotic fish of several centimeters dimensions that swim at high enough velocities to reach Reynolds numbers Re of order $10^3$ or $10^4$. Under confinement in circular arenas filled with a shallow layer of water, these robots swim mostly near the walls and undergo a gradual transition from swirling motion near the boundaries to large cluster formation as the number of particles in the assembly is increased. This transition is highly dependent on the nature of the walls: for solid impermeable walls this transition occurs for small numbers of fish robots. For porous walls this transition is delayed and occurs at larger numbers. The main reason why the two boundaries affect the swimming differently is the alignment of the fish robots at the wall: for the impermeable boundary the fish robots align with a smaller angle to the wall while for the porous case, the fish robots align with a larger angle at the wall allowing the formation of linear clusters. We carry out numerical simulations of model fish in three dimensions to examine how such experimental results can be understood. The interest of these simulations is that they provide a direct and quantitative view of the properties of the flow engendered by the fish like objects. The interaction of this flow with other fish or with the boundaries is the crucial aspect behind the self organization. These simulations reproduce the main features of the behavior of the swimmers such as their swimming near the walls or their angle with respect to the boundary. By using flexible and free to move arenas in experiments and simulations, we show that the assembly of fish robots is capable of creating large deformations as well as induce mobility of the arenas through the self-organization of the robotic fish opening the possibility of making sub-aquatic flexible robots of robots.

## Introduction

Interest in understanding the collective behavior of different animals and species (birds, fish, sheep, bacteria, etc...) is longstanding and different mechanisms for such behavior havebeen proposed^1–3^. An avenue to understand the physical basis of such behavior is to use model systems which consist of particles with mobility, and a few additional rules as to how they interact^1,2,4–6^. And indeed, several such models, both theoretical and experimental, are successful in finding collective dynamics and several features reminiscent of collective behavior such as schooling, flocking and swirling as seen in natural settings^7–10^. In this endeavor, much work has been carried out on artificial swimmers both theoretically and experimentally^4–6^. Several types of (artificial and natural) swimmers, which come in two major classes known as pushers and pullers, have been studied^6,11^. These are usually of microscopic dimensions and swim at Reynolds numbers close to zero. Much of this is of interest for bacteria and other microscopic entities such as light or chemically driven Janus particles. Most of these studies being at low Reynolds numbers, it remains a challenge to gauge how high Reynolds numbers affect the collective behavior of swimmers and what the effects of boundaries are for such conditions^12,13^. Whether this is of interest to the behavior of fish or other aquatic species in natural settings is an important question and a variety of experiments using different types of fish have been carried out revealing among others the role of social interactions, interactions with the environment, or the role of the flow produced by the fish in determining the behavior of the collective^12–20^. Experiments and simulations using artificial fish like swimmers should allow to shed light on the importance of hydrodynamics^12,21^ as well as the presence of boundaries on the behavior of collections of high Reynolds number swimmers.

In general, fish swim at a variety of Reynolds numbers ranging from $10^4$ to $10^7$^22–24^. Few experiments have considered the spatial organization of artificial swimmers with large inertial effects and thus at high Reynolds numbers. There are also very few model systems of artificial swimmers capable of such high Reynolds number swimming. As for numerical or theoretical models, almost none consider the high Reynolds number limit^25^. In principle and for numerical simulations a full fledged resolution of the Navier stokes equations with moving and possibly flexible entities are necessary. The other question, which remains without an answer, is the influence of boundaries on such behavior. For dry particles and low Re swimmers, it is now established experimentally that boundaries play an important role with the mobile entities (bacteria and small algae especially) accumulating or swimming near the boundaries. It is not clear again how the Reynolds number influences this trend.

Here we show that high Reynolds number artificial fish like swimmers, while displaying some features resembling low Re swimmers such as swimming near the boundaries as has been shown before^26^, exhibit some specific behavior. Even at small densities, the swimmers considered here have a tendency to swim close to boundaries and form stable clusters. These swimmers tend to form a robust swirling state at low and intermediate number densities. Our observations of the spatial organization of such fish in circular arenas, show that the nature of the boundaries affects the behavior of the fish in a nontrivial manner. These swimmers end up swimming along the boundary and in the same direction after a short period of time giving rise to swirling motion along the arena boundary. This swirling can persist as the number of fish increases only when the walls of the arena are made porous. For high enough number densities, this swirling ceases and is replaced (not abruptly) by the formation of cluster like domains where the fish remain parallel to each other but their swimming is hindered. Further, when the fish robots are placed in arenas that can deform and move, large deformations for very flexible arena walls and eventual locomotion of the arena enclosing a number of such robots can be observed. By taking advantage of the spatial organization of such swimmers in closed arenas, we show that such a collective behavior within the ’superstructure’ (arena and swimmers) gives it mobility and life of its own opening the possibility of making aquatic superstructures with non trivial properties (flexibility and locomotion). We corroborate these experimental observations by numerical simulations where swimmers are embedded in a layer of fluid and their dynamics obtained using direct numerical simulations of the Navier Stokes equations. These numerical fish have a beating frequency, through the beating of their caudal fin, which mimics that of the fish robots. The interest of these simulations is that they provide a direct and quantitative visualization of the mechanical properties of the flow engendered by the fish like objects. The interaction of this flow with other fish or with the boundaries is the crucial aspect behind the self organization.

## Results

**Fig. 1:**
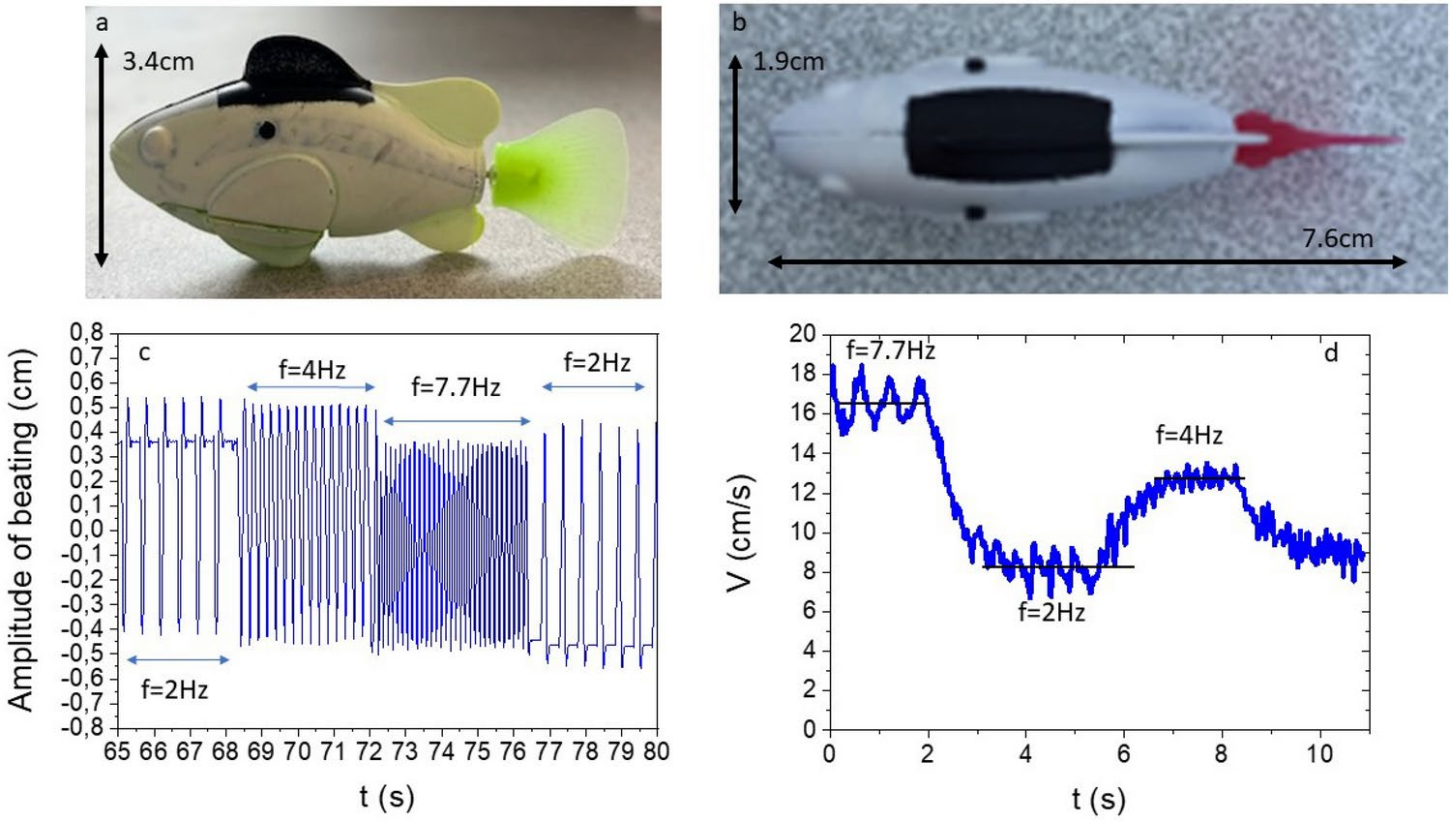
The fish robots: Photos of the toy fish (**a**) side view, (**b**) top view. (**c**) Beating amplitude versus time: three frequencies are apparent with the smallest one showing beating in one direction or the other (half duty cycle). (**d**) Velocity versus time. The velocity also shows three distinct values associated with the beating frequencies.

We start by focusing on a single swimming fish robot both in a large tank and in circular arenas. Then we characterize the effect of robot number on the organization of the assembly. We noted that the spatial organization of the assembly of fish robots is very sensitive to the type of boundary. We have used two different arenas, a glass cylinder and a porous one made of a net like boundary with pores of a few millimeters. The arenas are deposited in a larger tank with a water layer of a few centimeters in depth (5 cm) slightly higher than the height (3.4 cm) of the fish robot so that the fish swim without touching the bottom of the tank. The rationale behind the choice of a closed and porous boundaries is to act on the flow generated by the fish inside the arena in which they are enclosed. These entities, when swimming, create a large back flow in the enclosure. While the solid boundaries can dissipate this flow, it persists for long times nonetheless. The porous boundaries on the other hand allow this flow generated by the fish to escape the arena through the pores and into the surrounding water tank thus minimizing its effect. The other reason is that the fish can bounce somewhat on the walls of the arena. But we noted that this bouncing is more important for the solid glass boundary than for the net like softer boundary. Both effects can have important consequences on the spatial organization of the fish robots under confinement, effects we will study below.

### One fish

Our study focuses on fish like objects, Fig. 1 a and b, propelled by a caudal fin that oscillates at a frequency of several Hertz, see Fig. 1 c. The velocity of such fish can reach values of up to 15 cm/s, Fig. 1 d, giving Reynolds numbers ($Re=UL/\nu$ where *U* is the mean velocity of the fish, *L* its length and $\nu$ is the kinematic viscosity of the fluid used which is water) of approximately $10^4$. The making of these toy fish robots, which are commercially available, gives them the ability to change the beating frequency of their caudal fin in what seems to be a random pattern. This pattern, which lasts a few minutes (4 minutes roughly), then repeats several times while the fish is immersed in water. This gives the fish what looks like a random cruise velocity, at least for periods of time less than the repeat period of the frequency pattern.

The first characterization we carried out is the determination of the flapping frequency and amplitude of the caudal fin of the fish. From video recordings of the caudal fin when the fish is held fixed but immersed in water, we find that this frequency comes in three values: 7.7 Hz, 4 Hz, and 2 Hz. While the large and medium frequencies correspond to periodic back and forth beating with the fin going from left to right and back symmetrically, the smallest one corresponds to asymmetrical beating with the fin going from left to right to left followed by a quiet period or from right to left to right followed by another rest period. These two modes at low frequency allow the fish robot to turn right or left respectively. A plot of the beating amplitude of the end of the caudal fin versus time is shown in Fig. 1 c. The amplitudes of the three different frequencies are not exactly the same but they are all of roughly 1 centimeter. The different frequencies are clearly visible along with the half duty cycle ones.

**Fig. 2:**
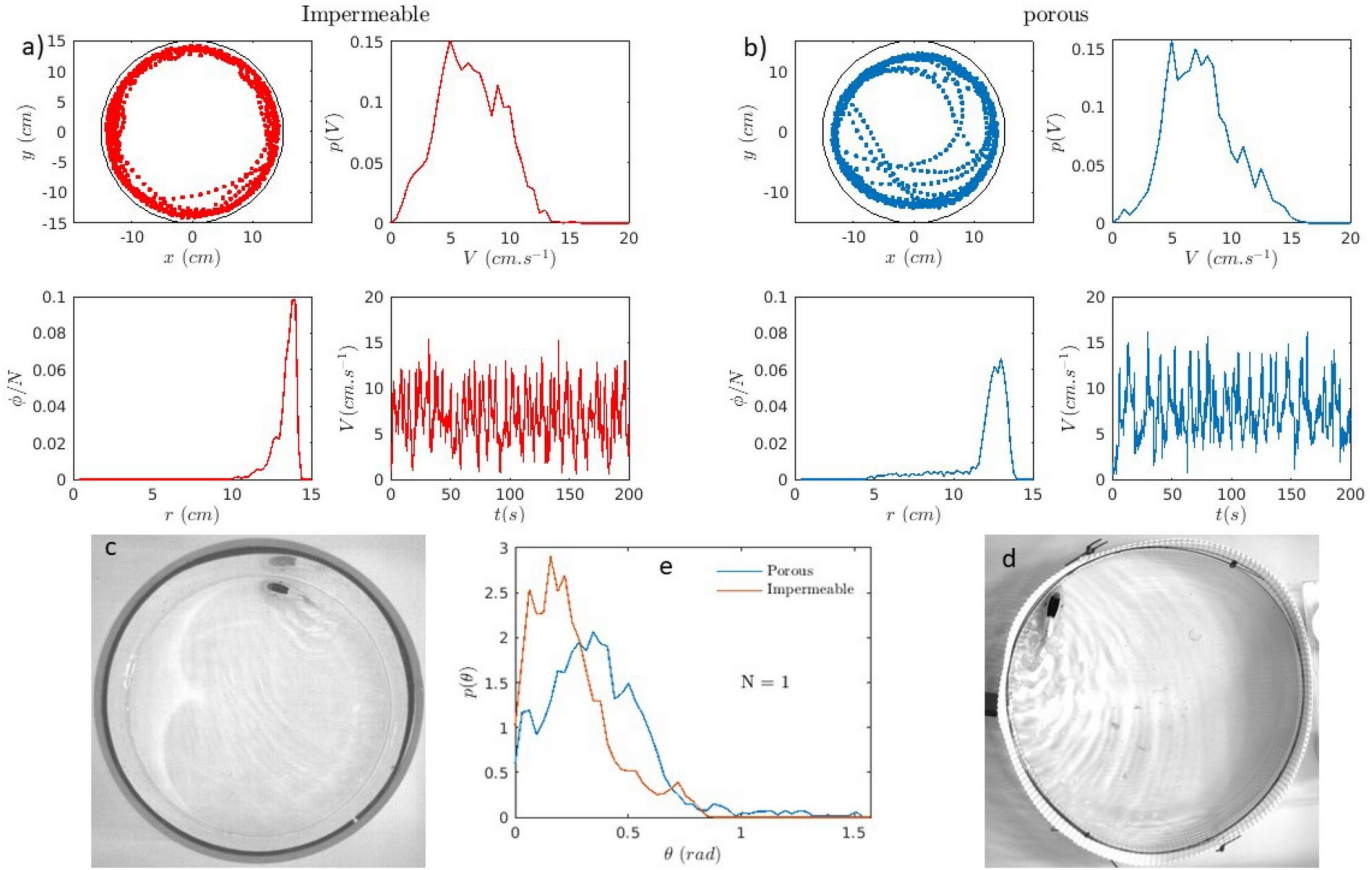
A single fish: (**a**) impermeable arena: The trajectories of the fish robot, instantaneous velocities and their histogram as well as the probability of presence are shown as red lines. (**b**) The trajectories of the fish robot, instantaneous velocities and their histogram as well as the probability of presence are shown as blue lines. (**c**) Photo of the fish robot in an impermeable arena. (**d**) Photo of the fish robot in a porous arena. Both arenas are embedded in a large water tank with a layer of water the height of which is controlled. (**e**) Probability distribution of the angle of the fish robot with respect to the wall for the two different arenas. The histograms are obtained from using runs of 304 seconds and 342 seconds for the impermeable and porous arenas respectively sampled at 7.5 Hz.

A single fish is then followed using particle tracking over a certain period of time, generally over 5 minutes in circular arenas but only 1 min or so far from any boundary in a large tank, to determine the fish position, its orientation, and its velocity. The fish have been painted with a black rectangle on their back, as can be seen in Fig. 1 a and b, so that their orientation and position can be obtained easily from black and white images of the arena. From such determinations, the instantaneous position of the fish can be followed over time. That the three frequencies are present is seen in the velocity time trace of a fish robot swimming in a large tank far from any boundary showing a fast velocity at near 17 cm/s, an intermediate velocity near 12 cm/s, and a smaller velocity near 8 cm/s, Fig. 1 d.

Next, we put a single fish in a circular arena and track its position versus time, Fig.2 a and b. This information is then used to extract the velocity time trace and velocity distribution as well as the probability of presence versus the radial distance from the center of the arena. Not surprisingly, the fish robot spends most of its time swimming along the boundary. It does escape from time to time but ends up returning to the wall area. The trajectory of the fish robot can be seen in Fig. 2a and b where most of the time, the fish robot swims along the arena wall.

The distribution of radial position obtained from this trajectory is thus peaked near the wall at roughly a fish robot width, and this for both arenas: for a glass cylinder and a porous plastic cylinder. From the histogram of velocity (total velocity), the distribution is wide but three different peaks at roughly 10–12 cm/s, 8-7 cm/s, and 5-4 cm/s seem to be apparent corresponding to the three different frequencies of beating of the caudal fin. These velocities are smaller than the ones of Fig. 1 where the fish robot was far from boundaries. The proximity of the boundary reduces the speed somewhat.

Overall, the fish robot swims near the boundaries over the course of their swimming. This feature is reminiscent of what occurs for low Reynolds number swimmers^26^or dry rod like particles^27–29^.

An interesting feature which distinguishes the two different boundaries is the angle of the fish robot with respect to the wall. As can be seen in the images of Fig. 2 c and d, the fish robot makes a smaller angle with the wall for the impermeable boundary compared to the porous boundary.

Figure 2 e shows the distribution of angles for the two arenas. While the distribution shows a peak centered at an angle of roughly $10^{\circ }$ or less for the impermeable case, the peak of the distribution is centered at a larger angle for the porous case. This angle is roughly $22^{\circ }$ in this case. As we will see below, this feature has direct consequences on the spatial organization of the fish in the two different arenas as we will see below.

### Increasing the number of fish robots in the arena

**Fig. 3:**
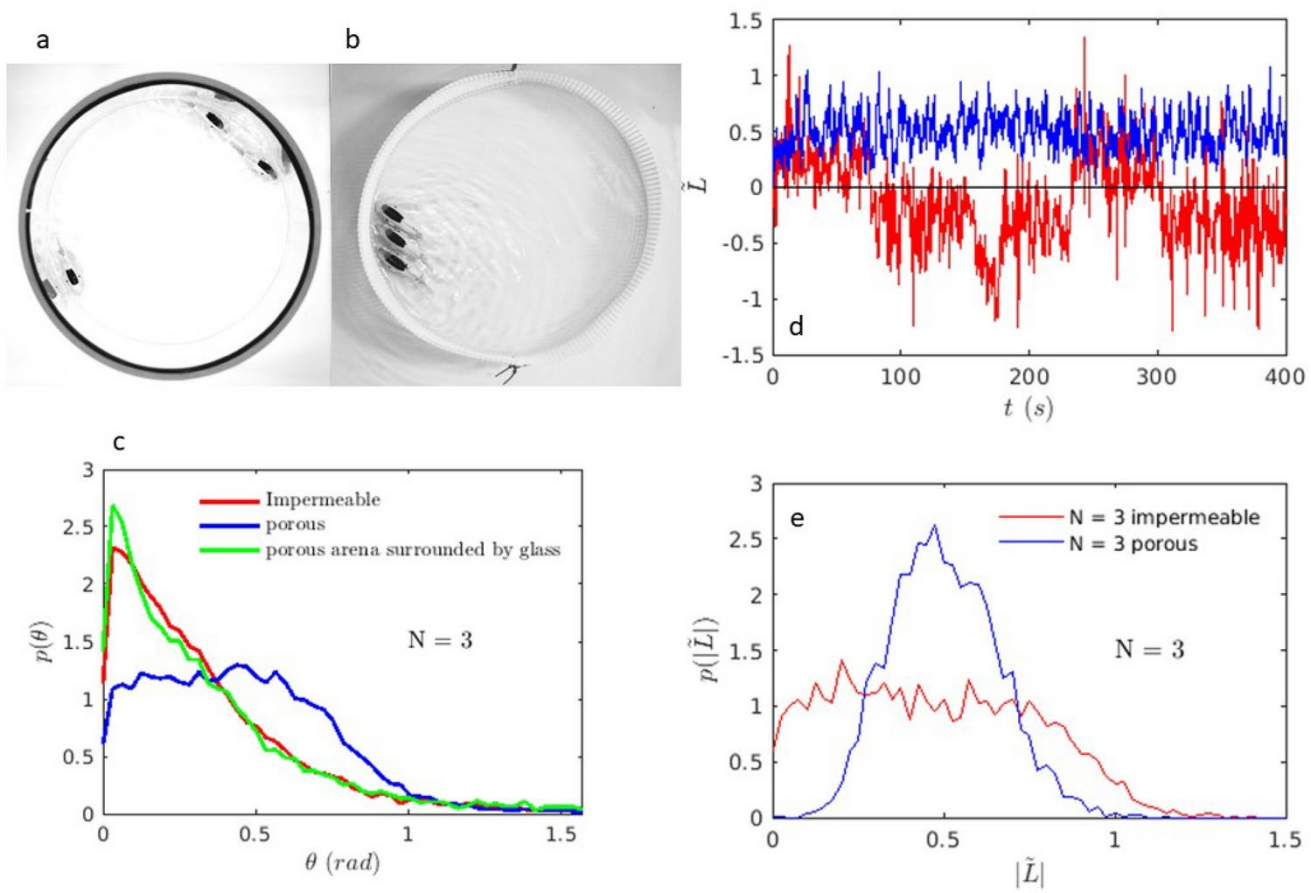
Three fish robots: Photos of three fish robots in the (**a**) impermeable and (**b**) porous arenas. Note that in the porous arena, the three fish robots form a stable cluster. (**c**) Probability distribution of the angle of the fish robots with respect to the walls in the two different arenas as well as in a porous arena surrounded by a glass boundary. These are obtained using runs of 738 seconds, 535 seconds and 563 seconds for the impermeable, porous, and porous surrounded by glass arenas respectively sampled at 7.5 Hz. (d) Time series of angular momentum and (**e**) angular momentum distributions obtained from the same runs as for the angles.

Let us now consider the role of the number of fish robots in the arena. As the number of robots increases, their spatial organization evolves. For a few fish robots, the robots continue swimming along the boundary and the radial distribution is peaked near the wall. While some features (radial distribution and velocity distribution) seem to be barely dependent on the boundary (solid or porous), as they remain roughly the same, an intriguing development occurs as can be seen in Fig. 3a and b for as little as 3 fish robots. Contrary to the impermeable boundary where no particular organization emerges apart from the fish robots swimming near the boundary and being more parallel to it (Fig. 3c), for the porous walls, the three fish robots self-organize rather quickly. They adopt a configuration where they all go around the arena in the same direction while maintaining a configuration where the three are parallel to each other and are at a slightly slanted angle with respect to the wall. This state of organization can persist for several minutes. If it breaks, the fish robots have a tendency to come back to this configuration rather quickly within tens of seconds. This is a direct consequence, we believe, of the angle at the wall of a single fish robot as we have observed above in Fig.2 e. This angle is roughly $22^{\circ }$ as can be seen in Fig. 3 c where the distribution of angles for the 3 robots is shown under different conditions.

For the glass arena, the three fish robots do not necessarily end up in the configuration described above. This configuration can occur, but does not last for long. Once it breaks, the fish robots take a long time before recovering this configuration with all fish robots swimming in the same direction. Generally, for the glass arena, the fish robots seem to make a small angle with respect to the wall and follow each other head to tail as seen in Fig. 3 a. The difference with the porous boundary stems from how the robots algn with the wall as seen in Fig.2 for 1 robot and Fig.3 c for the assembly of 3 robots where the most probable angle is close to zero for the impermeable cases.

An interesting and crucial observation is that the angle that the fish robots make with the boundary has a different distribution when the boundary changes. The fish robots are more parallel to the boundary for the closed one (glass impermeable boundary) but have a more tilted angle for the porous one. As the angle is more tilted, a configuration of 3 or more robots that are parallel to each other forms a small stable cluster as observed for the 3 fish robots above in Fig. 3 b. For the impermeable boundary, the fish robots make a small angle with respect to the the wall but they do not swim necessarily parallel to each other: they form a line of fish robots in a head to tail configuration where the follow each other, Fig. 3 a. Because of small collisions with the boundary and the fact that the fish robots can have different speeds, the robot ahead of the two others can bounce on the boundary and change course. As this happens, the second fish robot right behind it can overtake it and move upfront followed by the third robot. The two continue their way forward while the first one is pushed out of the boundary region and may change direction. The time for it to come back can be long. This difference, even at low fish robot number, has consequences on what occurs for a larger number of robots in the arena as we will see below. A test using a porous boundary surrounded by a glass cylinder seems to give results close to those obtained with the glass boundary (see Fig. 3 c) highlighting the effect of porosity instead of simply the presence of the pores and the associated roughness of the surface.

To distinguish among the different organizations and to further study the differences between porous and non porous walls, we calculate the normalized angular momentum of the assembly. This can be written as $\tilde{L}= \frac{\sum {\textbf {r(t)}}\textbf{x} {\textbf {V(t)}}}{VR}$. Here ${\textbf {V(t)}}$ is the velocity vector of the fish robot, ${\textbf {r(t)}}$ its radial position vector (from the center of the arena), *V* is the mean velocity of a single fish robot in a circular arena (which is 9 cm/s) and *R* the radius of the arena. $\tilde{L}$ is a vector but it only has one component in the vertical (or vorticity) direction since the layer is quasi two dimensional. The sum is carried out over all agents in the arena. This quantity is stochastic (see Fig. 3 d) as it is calculated for each image and its distribution is calculated for each configuration (porous or impermeable). If all fish robots are near and parallel to the boundary, and swim in the same direction, the value of $\tilde{L}$ is maximum with a value close to 1 (or −1). If the fish robots swim in different directions, or their positions are far from the wall, this angular momentum is smaller.

A time series of the angular momentum for 3 fish robots is shown in Fig. 3 d. Note that for the porous boundary, $\tilde{L}$ fluctuates around a nonzero value while for the impermeable boundary the value of $\tilde{L}$ can change sign and go through zero from time to time. The angular momentum (the absolute value of $\tilde{L}$) distribution for the 3 fish robots in the porous arena, Fig. 3 e, is peaked at a non zero value. Visual inspection shows that the 3 robots turn in the same direction and remain close to the wall which is consistent with the non-zero value of the angular momentum. We will refer to this behavior as swirling. Generally, the 3 fish robots would form a stable cluster with a finite angle with respect to the wall and swim in the same direction for a relatively long period of time as illustrated by the example of Fig. 3 b. However when the boundary changes to impermeable, the distribution of the angular momentum is no longer peaked at a finite non zero value. The distribution of the absolute value of *L*, Fig. 3 e, has a broad shape and an important component at zero angular momentum. In this case, swirling is less important with events where the fish robots no longer have the same direction becoming more and more important. This is the first sign that the boundary plays a role in how the fish robots self organize in the arena even for small numbers.

### Swirling state

**Fig. 4:**
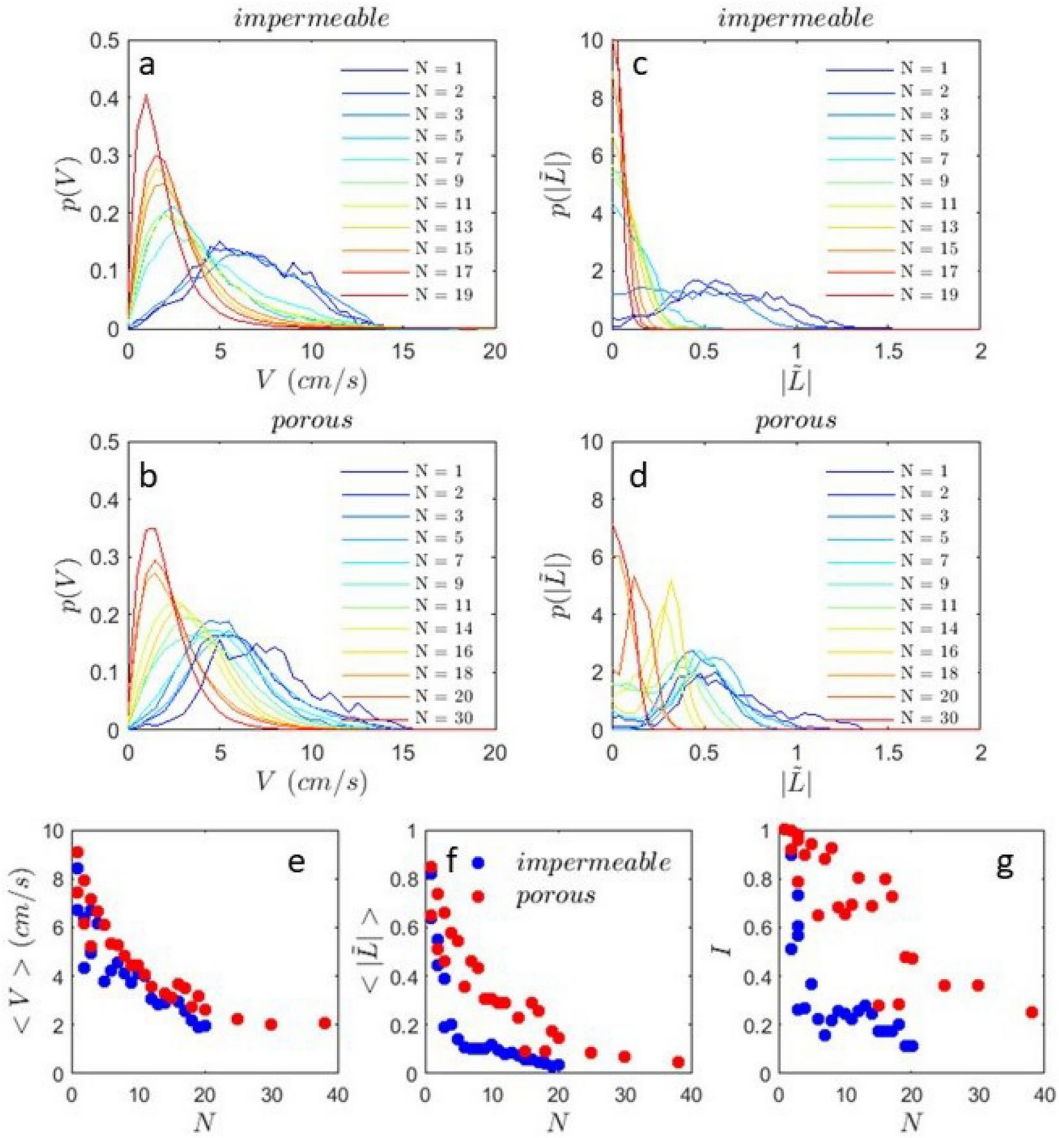
Many fish robots: Distributions of velocity (**a**) impermeable arena (**b**) porous arena. Angular momentum for (**c**) the impermeable arena and (**d**) porous arena. Different numbers *N* of fish robots in the arena are considered. (**e**) Mean value of velocity, (**f**) mean angular momentum and (**g**) proportion of robots swimming in the same direction *I* versus number of robots *N*. Blue symbols impermeable boundary, red symbols porous boundary. The runs last 350 seconds and are sampled at 7.5 Hz.

The distributions of velocity and angular momentum discussed above for a few fish robots are now examined for the two types of boundary for more robots. Figure 4 shows how these distributions vary with the number of fish in the same arena.

Consider the velocity distributions, Fig. 4a and b. In both arenas the velocity distribution is peaked at a velocity which depends on the number of fish *N*. While for small numbers (less or equal to 3) the shape and the peak position of the distribution do not change (for glass), the peak position starts to shift gradually to smaller and smaller values as *N* increases. For the porous walls a roughly similar phenomenology is observed. As the number of fish robots increases, a gradual slowing down of the robot assembly takes place independently of the nature of the boundaries.

Concerning the angular momentum, Fig. 4 c and d, as the number of fish robots increases, the distribution goes from a distribution with a peak far from zero to a distribution peaked around zero. The mean value varies thus from high values at low densities to close to zero at higher densities. This variation is more severe for the impermeable boundary where the distributions have a peak near zero as soon as the number of fish robots increases above 2 or 3. The distributions of $\tilde{L}$ for the porous boundary continue to have a peak away from zero for values of *N* as high as 18. Note that these changes occur while the velocity distributions remain roughly similar for the two arenas.

In Fig. 4e and f, we plot the mean value of the velocity and mean value of the angular momentum. The mean velocity decreases with the number of fish robots roughly similarly for the two types of boundary; the mean velocity is slightly smaller for the glass walls probably because friction is more important compared to the porous boundary where there is a certain amount of slip probably. Note that this decrease of the mean velocity with the number of robots is the hallmark of the mobility induced phase separation characteristic of active matter^30^ which we report here for for high Reynolds number swimmers.

The mean angular momentum variation versus *N* depends however more strongly on the type of boundary, Fig. 4 f. It decreases rather abruptly for the glass boundary at *N* close to 3 while the decrease is more gradual for the porous boundary leaving a wide range of number densities for which the angular momentum is larger for the porous boundary.

We will call the state with a non zero peak position for the angular momentum distribution, a swirling state. In this state most fish robots go around the arena in the same direction and swim close to each other. In Fig. 4 g, the proportion of fish robots swimming in the same direction remains high for the porous boundary while for the solid/impermeable boundary this proportion drops to zero very quickly ($N>3$). This is quantified by calculating $I=|(n^+-n^-)|/(n^++n^-)$ where $n^+$ or $n^-$ are the proportion of fish robots swimming in the clockwise or counter-clockwise direction. This quantity falls abruptly for the glass arena while it drops slowly and remains high up to almost 20 fish robots for the porous boundary.

**Fig. 5:**
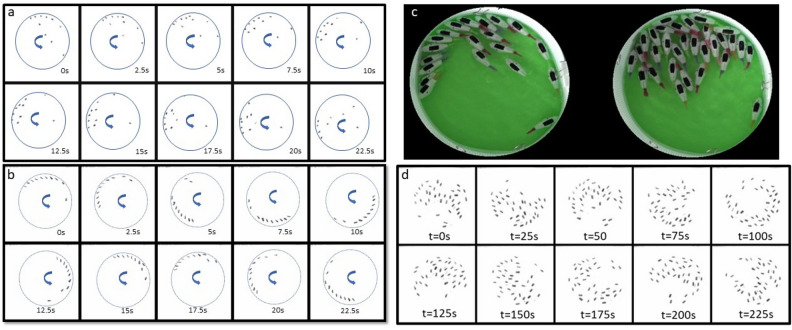
Spatial organization of fish robots: Time lapse images of robots in the two arenas (*N*=10 fish) (**a**) impermeable, (**b**) porous for an arena of 30 cm in diameter. Here only the black rectangular mark on the fish is visualized. Higher *N*: (**c**) Snapshots of fish robots in a 30 cm diameter porous arena for 20 fish in a linear cluster (left image) and a large number of robots (38 fish) forming a disorganized and static cluster (right image). (**d**) The images show a time series of 38 fish robots (only the black rectangle on the fish is visualized) in a disorganized state in a porous arena of 30 cm in diameter.

The most probable configuration of the fish robots for the high angular momentum state or swirling state seems to be the parallel configuration where the fish form a linear cluster with robots parallel to each other with a slight angle with the boundary as seen for the three fish robots above (Fig.3 b). For the glass boundary, the state of organization is more disordered with fish robots swimming in different directions giving rise to a smaller angular momentum.

While the swirling state with fish robots parallel to each other can persist for the porous boundary up to several fish, it does not for the solid boundary (apart from the trivial case of 1 and eventually 2 robots). The stability of the most probable configuration is thus highly dependent on the properties of the boundary with a more disordered state for the closed (glass) boundary. Note that this difference is related to the mean angle the fish robots make with the boundary. Figure 5 shows a montage of the fish robots configuration with the solid/impermeable (Fig. 5a) and porous (Fig. 5b) boundaries: Note that for the impermeable boundary the fish robots are more randomly placed and even if the group seems to travel roughly in the same direction (see arrow), the collective movement is very slow. For the porous boundary, on the other hand, the fish form a linear cluster where the fish are rather parallel to each other and are all at a slight angle with respect to the wall. This angle is roughly of $20^{\circ }$ as seen in the pdfs of the angle with the wall plotted earlier. The linear cluster allows the fish robots to undergo a global rotation as they all turn in the same direction (see arrow) with a full revolution in less than 20 s. Fig. 5 c (left photo) shows a snapshot of 20 fish robots most of which are in this configuration. For more robots and this is so for both arenas, the fish robots form disorganized clusters that barely move in the arena as can be seen in the snap shot of Fig. 5c (right photo) and time montage of Fig. 5 d for 38 fish robots in a porous arena of 30 cm diameter.

### Numerical simulations

**Fig. 6:**
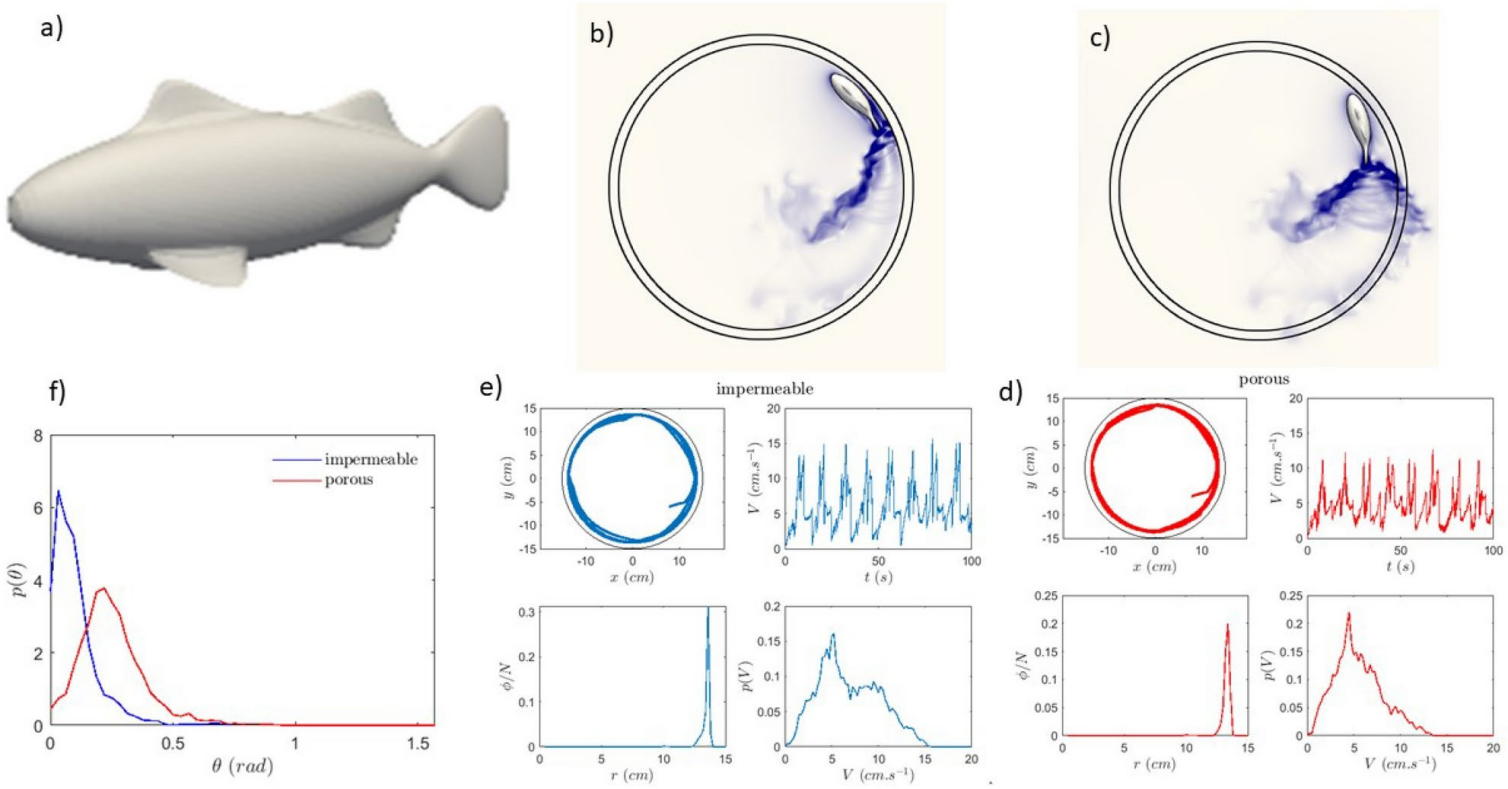
Numerical simulations for one fish: (**a**) Fish shape, and velocity field at the midplane of the arena for a single fish in (**b**) impermeable (**c**) porous arenas. The arena is 30 cm in diameter and the fish has the same dimensions (length, width and height) as the experiments. (**e**) and (**d**) Trajectory, velocity time trace, probability of presence of the fish and the pdf of the velocity for the two different arenas. (**f**) Histogram of fish angles with respect to the wall for the porous and impermeable boundaries. The runs lasted typically 180 seconds and are sampled at 1000 Hz.

To corroborate these experimental results, we have carried out direct numerical simulations (DNS) of the Navier Stokes equations of objects whose shape is similar or close to the fish robots used in the experiments^25,31^ and immersed in a shallow fluid layer, Fig.6a, b, and c. These simulations use the penalization method^32^ and both porous and impermeable walls are used. Further the shallow layer of fluid used is delimited by a solid bottom and a frictional top to account for the presence of the tank bottom and the free surface of the fluid. Also, these objects are propelled by the oscillations of the caudal fin which is programmed to mimic the experimental oscillations with roughly similar amplitudes and frequencies.

The incompressible Navier-Stokes equations, which describe the dynamics of viscous, incompressible fluids, are discretized in time using a fractional time-step method of the prediction-correction type, as proposed by Chorin and Temam^33,34^. This method is commonly used to solve the Navier-Stokes equations in a stable and efficient manner by splitting the velocity update into two steps: a first prediction step that computes a velocity field from a given pressure field, followed by a correction step to adjust this prediction and ensure the incompressibility and viscosity conditions are satisfied.

The spatial discretization is carried out using the finite difference method on a uniform Cartesian grid. This choice ensures ease of implementation while efficiently handling complex fluid problems. Convective terms, which represent the transport effects within the fluid, are discretized using a third-order upwind scheme, which is particularly well-suited for advection equations to avoid non-physical oscillations (such as ”overshooting”) and ensure better stability. Other terms, such as diffusion or pressure-related terms, are handled using second-order finite differences, providing a good balance between accuracy and computational cost. Some details can be found in references^25,31,35^.

In this simulation framework, immersed structures (such as swimmers) are modeled within the fluid. This requires a specific approach to handle the interaction between the fluid and these structures. The immersed boundary method^36^is used to represent these structures without explicitly modifying the mesh around them. This approach simplifies the computation while maintaining a good approximation of the boundary conditions imposed by the solid structures. This approach is coupled to the Volume Penalization approach to simplify the imposition of pressure boundary conditions on the solid boundaries^37^.

The swimmer’s surface is discretized using specific meshes, where forces and moments due to the fluid-structure interaction are calculated. These forces are then integrated over the swimmer surface to evaluate the swimmer’s dynamic behavior within the fluid. This includes the consideration of lift forces, drag forces, and other hydrodynamic forces that influence the swimmer’s motion. The method also accounts for changes in the shape of the swimmer in response to forces exerted by the fluid, allowing for simulations of complex interactions between the swimmer and the surrounding fluid^35^.

A key feature of this approach is the introduction of contact forces to handle collisions, both between swimmers themselves and between swimmers and surrounding walls. Managing these collisions is essential for simulating realistic interactions in environments where multiple solid structures may move independently and come into contact with each other. Lubrication and collision forces, developed in^38,39^, are employed to model the short-range interactions between swimmers, as well as between swimmers and walls.

The numerical solver has been validated through a series of varied test cases and practical applications, ensuring its robustness and accuracy. These tests include simulations of fluid-structure interactions as well as comparisons with analytical or experimental results when available^35,40^. The results demonstrate that the proposed method is capable of accurately capturing the effects of fluid-structure interaction, particularly in both low and high-speed regimes, and is able to handle complex geometries, such as those encountered in swimmer modeling presented in this study.

Figures 6 d and e show the results of the trajectory, velocity distribution, and probability of presence for a single numerical fish. Note that the fish remains near the wall for most of its trajectory as observed experimentally. The velocity time trace shows the different velocity regimes and the changes associated with the changing frequency of the beating of the caudal fin. The pdf of the velocity shows similar features as the experiments with a broad distribution and the presence of characteristic velocities associated with the three characteristic beating frequencies of the caudal fin. No major differences are observed for a single numerical fish between the porous and impermeable boundaries. A snapshot of these simulations is shown in Fig. 6b and c where the fish is near the boundary and the velocity field in the mid-plane of the shallow layer is shown. Note that for the porous boundary the flow field is present outside the arena as well. Note that a major difference between the two arenas is the wake behind the fish: one of the vortex streets behind the caudal fin is absent near the boundary for the impermeable one while both are present for the porous boundary. Note that these streets emerge when the caudal fin beats. The beating from right to left produces clockwise rotating vortices while the beating from left to right produces counterclockwise rotating vortices. This produces a reverse von Karman street^12^. Along these features we have determined the angle at the wall of the numerical fish and in this case a difference appears depending on the nature of the wall as we have observed in experiments. Figure 6 f shows the distribution of the angle the fish make with the wall. For the porous boundary, the distribution is peaked at an angle of roughly 0.22 radians (12/13 deg). This angle is compatible with the angle observed in experiments albeit smaller. For the impermeable boundary the distribution has a peak relatively close to zero (less than $6^{\circ }$) and many events very near zero as the fish is basically parallel to the wall. Both features are reminiscent of the experimental findings.

We believe that this difference in angle with respect to the wall is a consequence of the differences in the wake structure of the fish near the boundaries. As noted above, the caudal fin produces vortices when beating. These are produced near the tip of the caudal fin, and their direction of rotation depends on the beating direction. In fact the vortex street produced is referred to as a reverse von Karman street since the vortices behind the fin rotate in the opposite direction compared to a standard vortex street behind a static obstacle. These vortices can produce a drag on the tip as do wing tip vortices. This drag will alternate direction depending on the direction of rotation of the vortices. For the caudal fin stroke, the vortex will produce a force in the same direction as the direction of the stroke. Near the wall the fin beating towards the wall will experience a force pointing towards the wall. The absence of the vortices in the stroke away from the wall as observed for the impermeable boundary leaves a force imbalance where the tail is only pushed towards the wall giving rise to a small angle. For the porous boundary each stroke both towards the wall and away from the wall produces a force due vortices giving no net force on the tail. This difference in the flow details between the two boundaries gives rise to the differences in angle and as we have seen above to large differences in how the fish organize near the walls. The numerics give a fine and quantitative picture of the flow details and thus allow a physical understanding of the role of the boundaries and their nature on the behavior of the robo-fish.

**Fig. 7:**
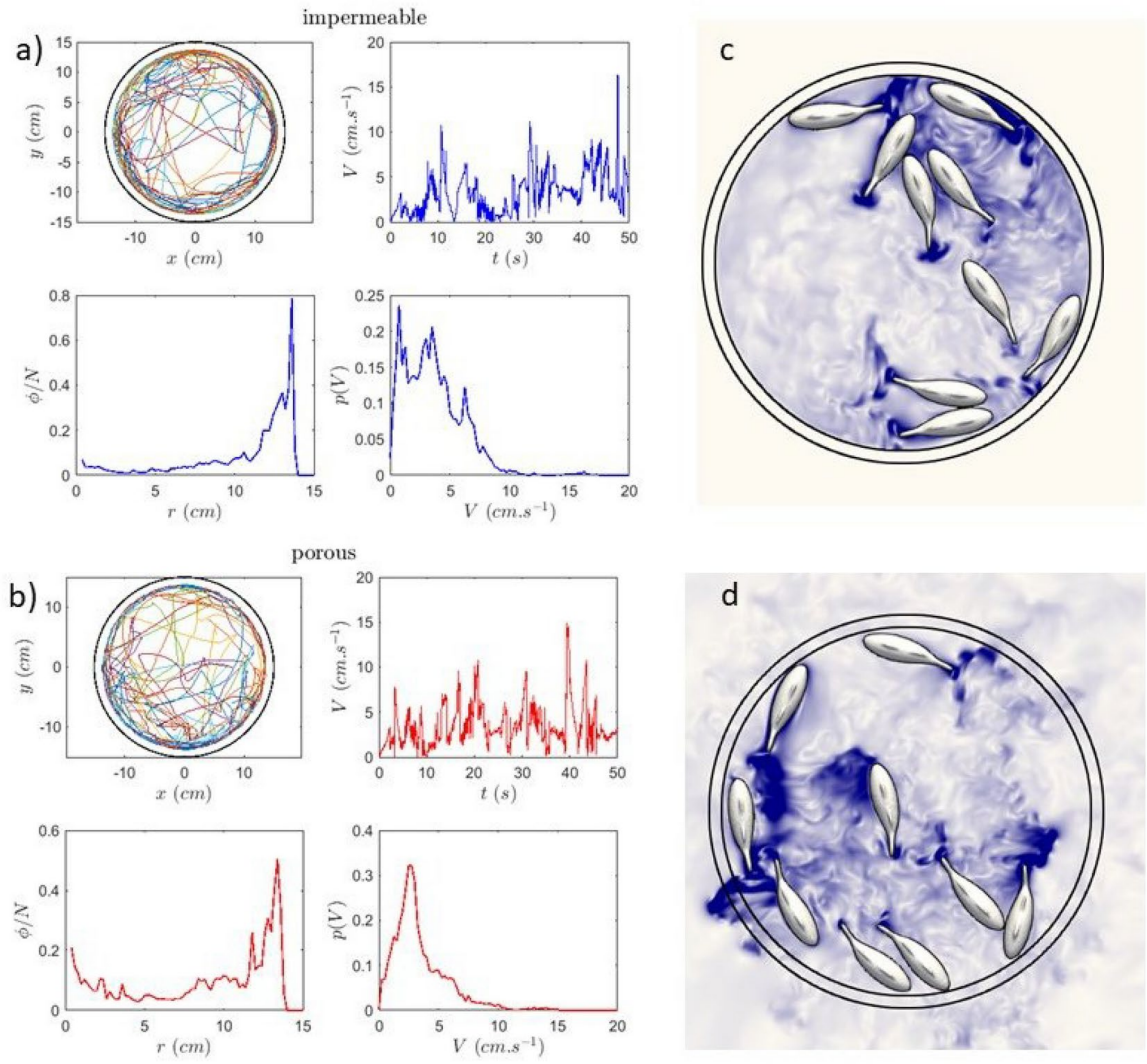
Numerical simulations for nine fish: (**a**) and (**b**) Trajectories (each fish has a specific color), velocity time series and histogram, and position distribution for 9 fish in the two different arenas. (**c**) and (**d**) Snapshots of the flow field (total velocity) at the mid-plane of the arena for 9 numerical fish in the impermeable and porous arenas respectively. The runs lasted 59 seconds and are sampled at 1000 Hz.

In a series of tests where 3 and 9 fish are used, different observations can be made. First of all, the distribution of the positions of the fish is basically constrained in a region very close to the wall as seen in Fig. 7 for nine fish. Second, the velocity distribution is broad but its peak shifts to smaller values as the number of fish increases as seen in comparing Figure 6 (e and d) and 7 (a and b) where the results for 1 and 9 fish are shown respectively. Apart from the differences in angle at the wall, it is difficult to distinguish between the two boundaries in the numerics as it is difficult to obtain enough statistics for the 9 fish case to see a difference in the angular momentum for example. For larger numbers of fish, the time duration becomes even smaller. The computational costs of this numerical simulation are considerable due to the extensive hardware requirements and large volume of data generated. A typical simulation runs for five days on a cluster of 256 CPUs, using a computational mesh with of 500x500x100 grid cells. While memory is not the primary limiting factor for the calculations themselves, the storage required to save the results is substantial. In particular, storing the output data - necessary for creating videos of the simulation - requires around 200 To per run, and sometimes even more to save the velocity and pressure fields as well as the fish positions allowing to generate the movies seen in the SI.

Nevertheless and considering the main features of the organization of the fish in the arena such as their probability of presence which is peaked near the walls and distribution of angles at the wall, as well as their velocity distributions, the numerics are in excellent agreement with the experiments. Further and even if the statistics are not sufficient, the velocity distribution shifts to smaller values as the number of fish increases again in very good agreement with experiments.

### Flexible mobile arenas

**Fig. 8:**
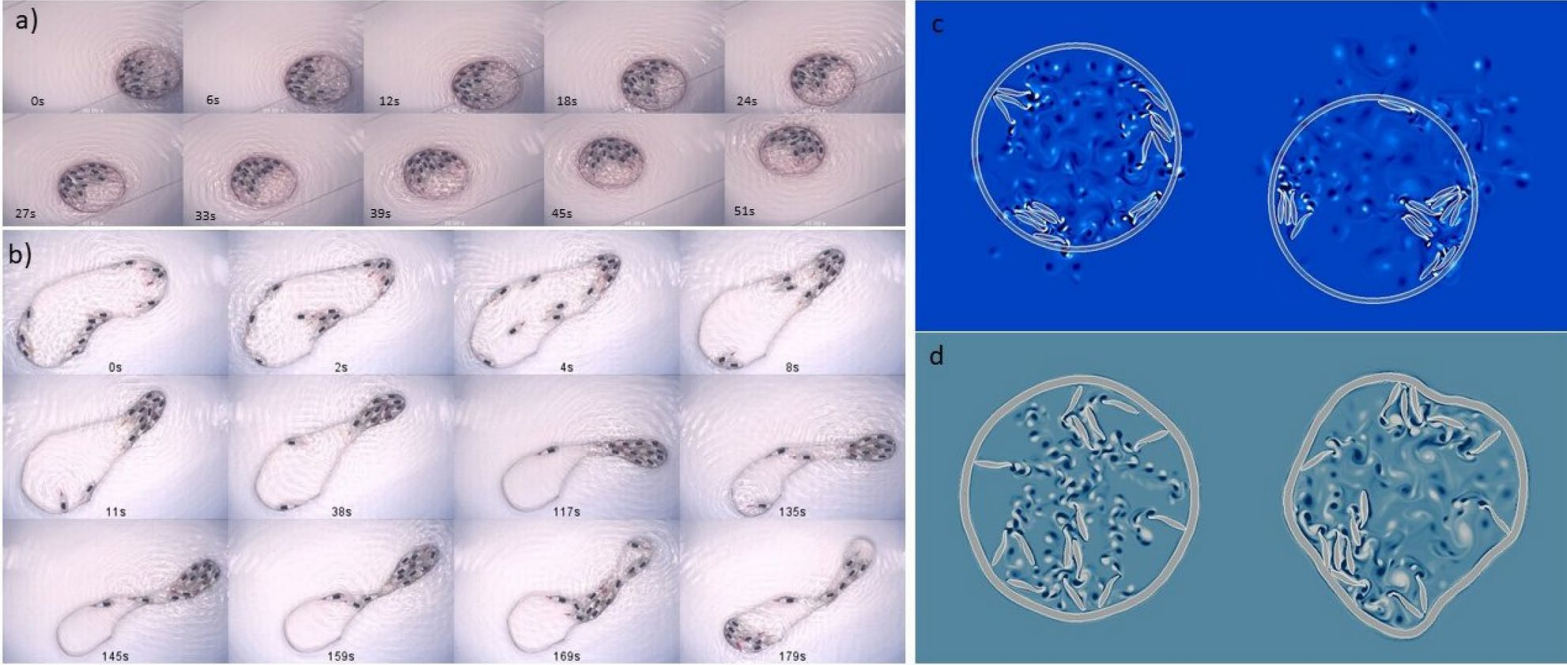
Flexible and mobile arenas: Experiments: (**a**) Twelve fish robots in a 9 cm radius arena which is free to move. Note that in tens of seconds the arena moves roughly two diameters. (**b**) Twelve fish robots in a 19 cm radius arena made of thin steel (100 microns in thickness making it very flexible). Note that the fish organize to pull the arena forward as well as change course. When the fish robots reorganize, the movement ceases. Numerics: Fifteen fish in 15 cm radius arenas which are free to move (**c**) or flexible (**d**). Note that the arena moves in the photos in (**c**) and deforms considerably in the photos in (**d**).

While in the results presented so far the arena was fixed and rigid, in an extension of this work we have used free to move and flexible arenas as shown in Fig. 8. In the first series of snapshots (Fig. 8 a) we show that the ensemble of fish robots may self-organize to exert a common push on the arena walls and push it forward over a distance of roughly two diameters in a matter of tens of seconds. In the montage Fig. 8 b, a much more flexible arena is used. Here again the fish robots self organize to exert a collective pushing on the walls both moving and deforming the arena. When the organization of the fish is perturbed the pushing ceases and new deformations start elsewhere.

Similar properties have been uncovered in other active matter systems both numerically and experimentally at different spatial scales giving the premises of flexible robotics based on collective dynamics at different scales both in aqueous or wet environments as well as dry ones^27,28,41,42^ We have simulated this behavior albeit for a 2D system as can be seen in the montages of Fig. 8c and d. The first montage shows a moving permeable arena and as it is permeable one can see the fluid flow generated by the fish leaking out of the arena. The second montage shows a flexible arena with the fish pushing on all sides and engendering large deformations. The simulations seem to capture the possibility that the fish may deform and move an arena or scaffold in agreement with the experiments.

## Discussion and conclusion

Our observations of the spatial organization of artificial fish in circular arenas of different natures, show that the presence as well as the nature of boundaries affects the behavior of the fish in a nontrivial manner. Even in small numbers, these swimmers end up swimming along the boundary, a feature which is reminiscent of low Reynolds number swimmers. They then end up swimming in the same direction after a short period of time giving rise to swirling motion along the arena boundary. This difference stems mainly from the orientation of the fish robots with respect to the wall: they align with a smaller angle with respect to the boundary for an impermeable wall compared with the porous boundary. We believe that this difference in orientation is a high Reynolds number effect as it is most probably due to how the nature of the boundary affects the wake behind the fish robot. The larger alignment angle for the porous wall favors linear clusters capable of swirling for long periods of time along the boundary. This swirling motion persists even for a larger number of fish robots for the porous boundary. Further, when the fish robots are placed in arenas that can deform and move, large deformations for very flexible arena walls and eventual locomotion of the arena enclosing a number of such robots can be observed. By taking advantage of the spatial organization of such swimmers in closed arenas, we show that such a collective behavior within the ’superstructure’ (arena and swimmers) gives it mobility and life of its own opening the possibility of making aquatic superstructures with non trivial properties (deformability, flexibility, and locomotion). We complemented these experimental observations by numerical simulations where swimmers are embedded in a layer of fluid and their dynamics obtained using direct numerical simulations. The interest of these simulations is that they provide a direct and quantitative visualization of the properties of the flow engendered by the fish like objects. The interaction of this flow with other fish or with the boundaries is the crucial aspect behind the self organization. Further work on the analysis of the flow structure and flow mediated interactions between fish and between fish and boundaries is necessary to further understand the observed phenomenology.

## Data Availability

All data is available upon reasonable request to the corresponding author.
